# Effects of a simulation-based blended training model on nurses’ treatment decision-related knowledge about oral cancer in Taiwan: a pilot survey

**DOI:** 10.3352/jeehp.2021.18.10

**Published:** 2021-05-25

**Authors:** Chia-Chang Huang, Shiau-Shian Huang, Ying-Ying Yang, Shou-Yen Kao

**Affiliations:** 1 Division of Clinical Skills Training, Department of Medical Education, Taipei Veterans General Hospital, Taipei, Taiwan; 2 Faculty of Medicine, National Yang-Ming Chiao Tung University, Taipei, Taiwan; Hallym University, Korea

**Keywords:** Mouth neoplasms, Nurses, Taiwan, Teaching materials, Virtual reality

## Abstract

The present study aimed to evaluate the effects of virtual reality (VR) simulations combined with bedside assignments on nurses’ self-efficacy in providing pre-treatment educational services. Between March 2019 and November 2020, we conducted a study of VR educational materials that were developed to cover information about the treatment of oral cancers. The effects of the VR simulation, the thinking-path tracking map method, and bedside assignments on the nurses’ treatment decision-related knowledge were evaluated in a ward for oral cancer patients at Taipei Veterans General Hospital, Taipei, Taiwan. The blended training model significantly increased nurses’ familiarity (P<0.01) and confidence (P<0.03) regarding their knowledge of treatments and treatment decision-related knowledge. This model also significantly increased their confidence in their skills in bedside pre-treatment education for admitted oral cancer patients (P<0.002). Oral cancer-specific VR materials enhanced the effectiveness of skills training among nurses in the oral cancer ward.

## Background/rationale

During hospitalization for cancer, primary nurses are the hospital staff who interact most frequently with anxious patients waiting for surgery, chemotherapy, or radiotherapy. Therefore, nurses are in a prime position to provide pre-treatment patient education, decrease patients’ anxiety, and increase patients’ satisfaction and adherence to treatment. Patients with early-stage oral cancer can be treated with oncologic surgery alone or in combination with an elective tracheostomy. In the advanced stage, patients can be treated with radiation therapy, chemotherapy, and immunotherapy, as well as combinations of those modalities with other treatments as necessary [1]. Compared with patients with other types of cancers, patients with oral cancer and their family members need more physical, psychological, and social support because of unresolved pre-treatment, peri-treatment, and post-treatment cosmetic distress.

Patients’ degree of understanding and recall of detailed medical information provided by the treating physicians and primary care nurses affects their adherence to forthcoming treatments. Unfortunately, patients with limited medical background knowledge remember as little as one-fifth of the information discussed and immediately forget 40%–80% of the content of their medical encounters. In particular, anxious patients recall very little of the medical information provided by healthcare providers and may be unsatisfied with the medical information that they receive [2]. Therefore, audiovisual educational aids have been suggested as a way to provide comprehensive pre-treatment education for patients with cancer.

Virtual reality (VR) technology can create ideal educational aids that provide consistent, vivid, and real-time interactions and immersive experiences. VR-based training materials are considered to be an effective tool to supplement in-person education and to cover a variety of topics [3]. In particular, VR materials can increase users’ motivation and engagement, as well as improving their health knowledge [4,5]. Patients undergoing cancer treatment perceive nurses as individuals who can reduce their anxiety by providing treatment decision-related information through audiovisual aids [6]. In light of patients’ requests for information on treatment decisions, nurses need to be familiar with and have confidence in providing patient-tailored information using interactive educational aids. A baseline survey at our institution revealed deficiencies in nurses’ familiarity with and confidence in their knowledge of treatment decisions and details of treatments, including surgery, radiotherapy, chemotherapy, immunotherapy, and molecular-targeted therapy.

## Objectives

This study aimed to evaluate the effects of VR-based training including the thinking-path tracking map method, VR simulations, and bedside assignments on the familiarity, confidence, and anxiety regarding treatment decisions and treatment-related knowledge and service skills of nurses in an oral cancer ward in Taiwan.

## Ethics statement

Ethical approval was granted by the ethics committee of Taipei Veterans General Hospital (IRB approval no., 2018-07-030AC and 2019-12-007ACF). All of the nurses who were invited to participate were informed about the value of the educational intervention and written consent was obtained. Nurses were informed that their regular training would not interfere with their participation in the study.

## Study design

This was a pre- and post-intervention comparison study.

## Setting

This study was conducted from March 2019 to November 2020 at Taipei Veterans General Hospital in Taiwan, which provides a high quality of medical care for oral cancers.

## Participants

This study was conducted among 26 primary nurses in the oral cancer care unit of Taipei Veterans General Hospital.

## Variables

**Fig. 1.**
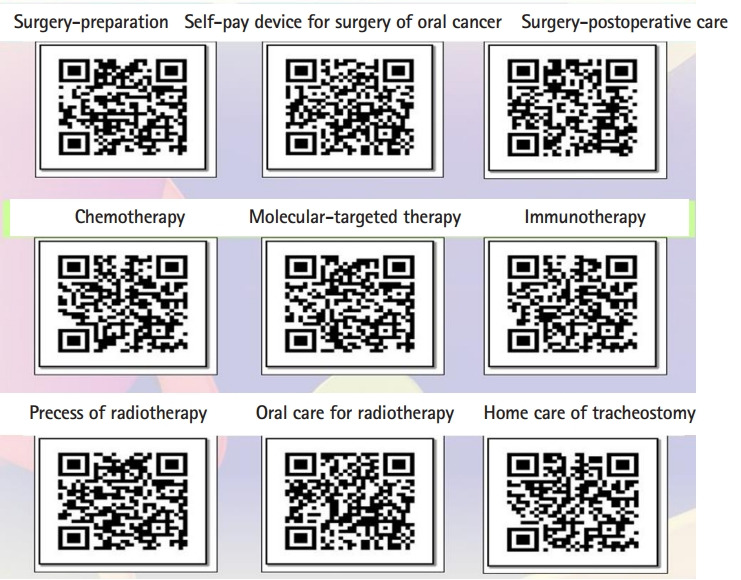
QR code of video of the virtual reality materials for pre-treatment education of oral cancer patients in Taiwan.

Using the questionnaire in Table 1, preceptors in this study evaluated oral cancer primary nurses’ familiarity, confidence, and anxiety regarding treatment decisions and treatment-related knowledge and service skills at baseline and after VR-based training. It was the 5-points Likert scale. Besides Table 1, the age and target training video was also obtained from 9 materials in Fig. 1 presented as QR code.

## Data sources/measurement

**Fig. 2.**
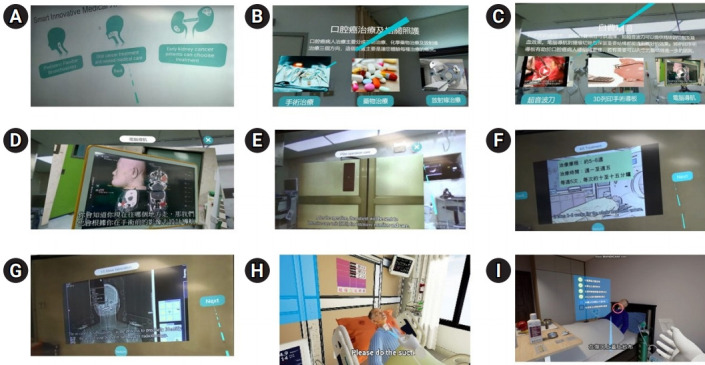
Representative images of our self-developed 3-dimensional (3D) and 4-dimensional virtual reality (VR) educational materials containing detailed treatment information for oral cancer for training primary nurses. (A, B) Introduction to 3D printed materials, computed tomography-guided operation, use of a harmonic scalpel for hemostasis, and postoperative precautions after surgery for oral cancer. (C) Introduction to care skills for patients who receive elective tracheostomy. (D) Introduction to the process of localizing the area for radiotherapy, making individualized shielding, creating the treatment schedule of radiotherapy, taking peri-treatment precautions, receiving post-radiotherapy outpatient follow-up, and carrying out oral care during radiotherapy. (F) Introduction to chemotherapy, molecular-targeted therapy, and immunotherapy. (E, G) Acceptable or unacceptable behaviors during chemotherapy and radiotherapy were taught by asking nurses to interact with the VR game, and the VR app responded to nurses about whether their choice of acceptable behaviors was correct. Simulated patients in (H) headset- or (I) smartphone-displayed VR content.

In the development phase, specific all-in-one educational materials with detailed information about treatments for oral cancer were created. In the implementation phase, nurses in oral cancer units were trained to provide bedside pre-treatment education with VR materials. Information on surgery included surgical preparation, postoperative care, and home care for tracheostomy. For radiotherapy, patients experienced simulations of the processes of localizing the area for radiotherapy, making the treatment schedule of radiotherapy, taking peri-treatment precautions, receiving post-radiotherapy outpatient follow-up, and carrying out oral care. A detailed introduction to chemotherapy (pre-chemotherapy preparation, side effects, schedules, and precautions), molecular-targeted therapy, and immunotherapy were also included (Fig. 2).

**Fig. 3.**
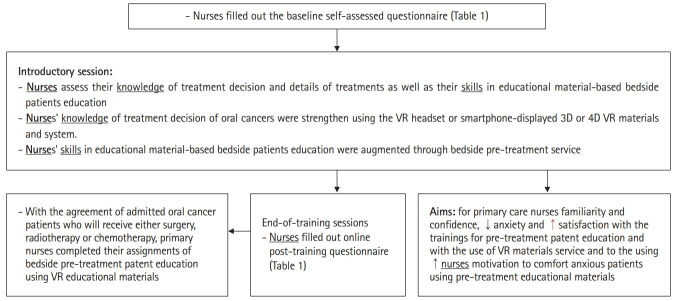
Flow chart of the blended-type training module of nurses for providing pre-treatment information to patients with oral cancer about their forthcoming treatment using the all-in-one virtual reality (VR) educational materials in the oral cancer ward. 3D, 3-dimensional; 4D, 4-dimensional.

To increase nurses’ engagement, acceptable or unacceptable behaviors during radiotherapy and chemotherapy were taught by asking nurses to interact with the VR game. Specifically, the VR app responded to nurses regarding whether their choices were correct. The training program emphasized improving nurses’ treatment decision-related knowledge and skills in presenting educational materials. In the introductory session, every part of the VR educational material was introduced to nurses through the VR headset. After an introduction to the goals of the training project (Fig. 3), the nurses then took turns experiencing each part of the headset-displayed VR educational materials. The nurses assessed their self-efficacy before and after the training session using the questionnaires in Table 1. In the clinical staging-specific thinking-path map test, nurses received a knowledge score by drawing a decision path on the printed algorithm of treatment according to the clinical stage in accordance with the clinical stage of each patient with oral cancer with whom they completed their bedside assignment.

## Validity and reliability of the measurement tool

The Cronbach α reliability test, as a measure of the internal consistency of the questions in Table 1, was 0.63 (details in Dataset 1). This result indicates that the internal consistency of the questions needs to be improved in a future study. Upon an evaluation by 3 experts, the content validity index of the 7 questions in Table 1 ranged from 0.8 to 0.89, indicating that the experts considered the relevance of the questions for the training objectives to be excellent.

## Study size

The sample size for this before-after study using the paired t-test was calculated using http://statulator.com/SampleSize/ss2PM.html. With an expected mean±standard deviation of the paired differences of 10±15, type I error (significance) of 0.05 and type II error (1-power) of 0.2, the estimated sample size was 24. This pilot study enrolled a total of 26 primary nurses from the unit for oral cancer patients.

## Statistical methods

Statistical comparisons of baseline and post-training (or post-education) data were made using the paired Student t-test. All statistical analyses were performed using IBM SPSS ver. 21.0 (IBM Corp., Armonk, NY, USA) and P-values <0.05 were considered to indicate statistical significance.

## Demographic information of participants

**Fig. 4.**
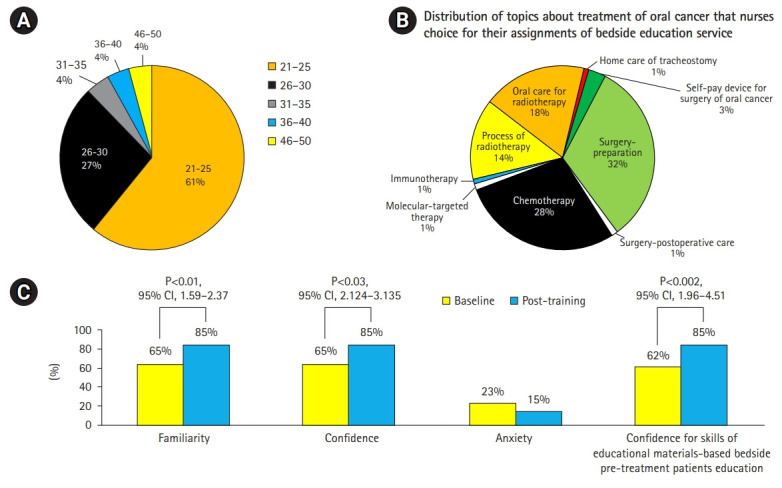
Characteristics of the nurses who participated in this study. (A) Distribution of the age range of the enrolled nurses. (B) Distribution of the topics that nurses chose to provide patients with oral cancer as part of bedside pre-treatment education according to each patient’s needs. (C) Comparison of the percentage of nurses who responded “strongly agree” or “agree” to the individual questions in the questionnaire evaluating baseline and post-training familiarity, confidence, and anxiety regarding knowledge about treatment decisions and the details of treatments. CI, confidence interval.

As shown in Fig. 4A, most of the participants were 21–30 years old. Two of the 26 nurses were men and 24 were women. Five nurses (19%) had more than 3 years of work experience in the oral cancer ward, 13 (50%) had 2–3 years of experience, and 8 (50%) had less than 2 years of experience.

## Main results

### Development of all-in-one VR materials and bedside assignments increased nurses’ self-efficacy regarding their knowledge and skills in pre-treatment education

According to the needs of each patient with oral cancer for their forthcoming treatment, the most common topics that nurses chose for their bedside assignment were preparation for surgery (8), chemotherapy (7), oral care for radiotherapy (5), process of radiotherapy (3), immunotherapy (1), molecular-targeted therapy, (1) and home care of tracheostomy (1) (Fig. 4B). At baseline, most of the nurses had a low level of familiarity with and confidence in their pre-treatment patient service-related knowledge and skills in providing bedside services (Fig. 4C). In comparison with the baseline values, the post-training scores showed increased familiarity and confidence, as well as decreased anxiety regarding their knowledge of treatment decision-making and details of treatments (Fig. 4C). Post-training improvement was also found in nurses’ confidence in their skills in educational aid-based bedside patients education.

### The increase in treatment-decision knowledge scores of nurses was associated with their positive responses to the newly developed all-in-one educational materials

**Fig. 5.**
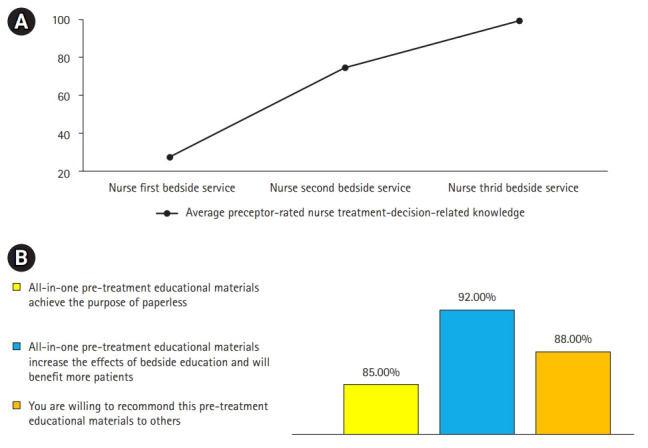
The increase in nurses’ scores for knowledge about treatment decisions was associated with their positive responses to the newly developed all-in-one educational aids at the post-training stage. (A) Changes in nurses’ scores for treatment-decision-related knowledge across their first, second, and third experiences of providing education to patients. (B) The percentage of nurses who reported that they strongly agreed or agreed that the all-in-one virtual reality educational aids achieved the purpose of paperless materials, increased the effects of bedside pre-treatment education and would benefit more patients, and were willing to recommend it to others.

In this study, every nurse completed 2–3 assignments of bedside patient pre-treatment education. Fig. 5A reveals that the average preceptor-rated scores for treatment decision-related knowledge increased progressively across the first, second, and third bedside service. After training, a high percentage (85%–92%) of nurses reported that they strongly agreed or agreed that the VR educational materials achieved the purpose of paperless format, strengthened the effects of bedside patient education and would benefit more patients, and were willing to recommend it to others (Fig. 5B).

## Key results

This study revealed a baseline lack of appropriate materials, a deficiency in nurses’ treatment decision-related knowledge, and inadequate skills in bedside pre-treatment educational services for patients with oral cancer. These deficiencies may be related to inadequate training during their undergraduate years as well as a lack of ongoing post-graduation workplace training and practice sessions. After VR training, increased treatment-decision knowledge scores were observed, which were associated with their positive responses to the newly developed all-in-one educational materials.

## Interpretation

In the limited time of pre-admission medical encounters, without supplemental audiovisual materials, healthcare providers often fail to provide patients with sufficient medical information about treatments [7]. Primary nurses need to be competent in using disease-specific audiovisual materials to provide complete and accurate pre-treatment information to anxious patients [8]. In our study, the all-in-one VR educational materials for patients with oral cancer were well accepted by nurses. Overall, the nurses responded positively to virtual simulation training and the all-in-one VR materials.

This VR service model combined medical knowledge and modern technology and transformed complex and abstract medical knowledge into an acceptable VR format that helped nurses convey the information easily and precisely. The results of this study may reduce patients’ degree of anxiety and frustration. In the future, this service could be expanded to other medical subspecialties and other hospitals.

## Comparison with previous studies

It has been reported that patients’ satisfaction and information recall were higher after receiving services provided by a more clinically competent medical team [9]. Therefore, communication training of the medical team is necessary to improve information recall and patients’ adherence. The baseline survey in our study highlighted the need to arrange regular workplace training and practice sessions among nurses in oral cancer care units. According to a recent survey report on the utility of smartphones, the prevalence of smartphone use is as high as 70.4% [10], of which aim was to create a smartphone-based service model to provide healthcare information through VR technology assistance. The positive results of present study may help to expand the usage of VR services to the Google App Store or Apple Store so that the service can be provided to a larger population in need of such services.

## Limitations/generalizability

This study has the limitation of a small sample, the potential impact of the timing of the intervention, and the assessment. The findings of this study suggest that reviewing medical records, holding discussions with physicians, participating in VR-based programs to strengthen treatment-decision knowledge, and engaging in bedside practice are feasible strategies to prepare healthcare professionals in the general ward setting to offer comprehensive pre-treatment services for oral cancers or other diseases by using web-based VR or written educational aids, thereby improving the quality of care.

## Conclusion

In this study, nurses in the oral cancer ward offered positive responses to the training and newly-developed written and VR materials for oral cancer treatment. The results of the current study highlight the importance of VR-based training, including nurses’ thinking-path tracking map method, VR simulation, and bedside assignments, on the knowledge and skills of primary nurses in an oral cancer ward. The initial results of our study encourage further application of VR materials in workplace training sessions for primary nurses caring for oral cancer patients.

## Figures and Tables

**Table 1.** Survey on nurses’ self-assessed self-efficacy and satisfaction

	Questions
Baseline****self-efficacy	1. I am *familiar with* **knowledge** about treatment decisions and the details of treatments.
	2. I am *confident* in my **knowledge** of treatment decisions and the details of treatments.
	3. I am not *anxious* about my **knowledge** of treatment decisions and the details of treatments.
	4. I am *confident* in my **skills** in educational material-based bedside patient education.
Post-training self-efficacy and satisfaction	1. I am *familiar with* **knowledge** about treatment decisions according to the clinical staging of oral cancer patients.
	2. I am *confident *in my **knowledge** of treatment decisions according to the clinical staging of oral cancer patients.
	3. I am not *anxious* about my **knowledge** of treatment decisions according to the clinical staging of oral cancer patients.
	4. I am *confident* in my **skills** in educational material-based bedside patient education.
	5. The all-in-one pre-treatment educational material was effective in a paperless format
	6. The all-in-one pre-treatment educational material strengthened the effects of bedside education and will benefit more patients.
	7. I would recommend these all-in-one pre-treatment educational materials to others

All questions were responded to using 5 choices: 5 (strongly agree), 4 (agree), 3 (disagree), 2 (strongly disagree), and 1 (not applicable).

## Footnotes

**Data availability** Data files are available from Harvard Dataverse: https://doi.org/10.7910/DVN/06HBPP Dataset 1. The data file contains the currently-used data, internal consistency (reliability) and content validity of the data that are presented in the manuscript.

## Supplementary materials

Supplementary files are available from Harvard Dataverse: https://doi.org/10.7910/DVN/06HBPP

Supplement 1. Audio recording of the abstract.
